# Phenotypic Characterization of ALS-Causing SOD1 Mutations Affecting Polypeptide Length

**DOI:** 10.1155/humu/9792233

**Published:** 2025-06-16

**Authors:** Mariusz Berdyński, Krzysztof Safranow, Peter M. Andersen, Cezary Żekanowski

**Affiliations:** ^1^Department of Neurogenetics and Functional Genomics, Mossakowski Medical Research Institute, Polish Academy of Sciences, Warsaw, Poland; ^2^Department of Biochemistry and Medical Chemistry, Pomeranian Medical University, Szczecin, Poland; ^3^Department of Clinical Sciences, Neurosciences, Umeå University, Umeå, Sweden

## Abstract

**Background:** Some 234 mutations in the small *SOD1* gene have been reported to cause amyotrophic lateral sclerosis. However, the pathogenic mechanisms, particularly of those mutations affecting polypeptide length, are contested. It is presently unknown whether all reported nonsense mutations in SOD1 are causative for ALS. The emergence of promising new anti-SOD1 drugs has made it imperative to gain further insight into clinical–genetic aspects of ALS for deciding which patients to treat in clinical practice and include in drug trials.

**Objective:** This study is aimed at comprehensively analyzing the clinical phenotypes associated with ALS-causing SOD1 mutations that alter the polypeptide length. The specific focus is on the age at which symptoms manifest and the survival duration.

**Methods:** Data were collected from web databases, published reports, conference presentations, and personal communications up to November 2023. The clinical endpoints, including age at symptom onset and age at death, were subjected to survival analysis. Comparative analyses were performed between frameshift and nonframeshift variants.

**Results:** A cohort of 146 ALS patients harboring 38 different nonmissense *SOD1* variants was analyzed. The mean age of disease onset was 46.9 years, with a mean survival duration of 49 months. Significant heterogeneity was observed in clinical outcomes, with earlier disease onset and reduced survival associated with specific mutations. Notably, frameshift mutations proximal to the N-terminus showed a higher risk of early ALS onset compared to more distal mutations.

**Conclusions:** The clinical phenotypes of ALS patients with nonmissense SOD1 mutations are highly variable and dependent on the specific mutation. These findings underscore the necessity of including diverse *SOD1* mutation carriers in therapeutic trials and suggest that both loss-of-function and gain-of-function mechanisms may contribute to ALS pathology.

## 1. Introduction

### 1.1. Mutations Causing Amyotrophic Lateral Sclerosis (ALS)

ALS is a heterogeneous neurodegenerative syndrome leading to muscle weakness and eventually atrophy. In Europe, the median incidence rate is 2.08 per 100,000 population (1.47–2.43), corresponding to 15,355 (10,852–17,938) cases [1]. In the United States, for 2018, the National ALS Registry found 21,665 persons meeting the case definition for a prevalence of 6.6 per 100,000 persons [2]. While 1%–10% of patients autoreport a familial predisposition for ALS (fALS), screening of ALS gene panels has revealed that at least 20%–36% of all patients irrespective of reported medical history carry a claimed pathogenic variant in one or more ALS genes. fALS is inherited in an autosomal dominant or recessive mode, frequently with reduced penetrance, and pleiotropy has been demonstrated for mutations in at least three genes [3]. In 1993, missense mutations in the small gene encoding the free radical scavenging enzyme Cu-Zn-SOD (SOD1) were linked to causing fALS in 13 of 18 studied pedigrees with high-penetrant ALS in multiple generations [4]. This sparked a global screening for mutations in *SOD1* and since then, some 234 mutations have been claimed to cause ALS. Though SOD1 mutations are rare in some seemingly similar populations (e.g., prevalent in the United Kingdom, rare in Ireland) in many countries mutations have been found in 7%–23% of diagnosed fALS cases but also in 3%–7% of sALS cases [5–7].

SOD1, a homodimer containing copper and zinc ions, is expressed constitutively at a high level, with differences between tissues and cell types, and can be detected in most subcellular compartments including cytoplasm, in the mitochondrial intermembrane space, nucleus, and endoplasmic reticulum [8–11]. SOD1's established role is to scavenge superoxide free radicals generated in the normal cell metabolism to hydrogen peroxide and water. Other reported functions include involvement in ferroptosis, as a transcription factor, a regulator of RNA stability, or a component of nutrient signaling [12–14]. SOD1 is ubiquitously and constitutively expressed in all cells in the human body and accounts for up to 1% of total cytoplasmic proteins in some areas of the CNS [15].

Although the SOD1 protein has been extensively studied, the pathomechanism by which SOD1 mutations cause ALS remains elusive. Originally, SOD1 mutations were proposed to cause ALS via a loss-of-function mechanism, but currently, the gain of function mechanism is favored. Several mechanisms have been proposed to cause the disease upon SOD1 inactivation: excitotoxicity, oxidative stress, ER stress, mitochondrial dysfunction, axonal transport disruption, prion-like propagation, and noncell autonomous toxicity of neuroglia [16]. Since 1993, over 200 different *SOD1* mutations distributed over the entire gene have been described, mostly missense, with quite a few mutations affecting the SOD1 polypeptide length (e.g., nonsense, insertion, deletion, and splice site variants) [17]. Most SOD1 “structural” variants are not deemed pathogenic by the American College of Medical Genetics and Genomics (ACMG) standards (similarly to the missense ones); moreover, for many of them, loss-of-function ALS mechanisms have been proposed.

This study is aimed at analyzing the phenotypic characteristics of the ALS-causing SOD1 mutations that affect the polypeptide length with a particular stress on the ages at symptom onset and death.

## 2. Methods

We collected all available data from individual or family ALS case reports, primarily from the ALS Online Database (https://alsod.ac.uk) [18] and published reports (PubMed search, conference reports, or personal communications, up to November 2023).

Analyses include only published data such as sex, clinical phenotype, age at symptom onset (age of onset or last known age without ALS symptoms), age at death, and familial or sporadic inheritance of patients with individual mutations (see Supporting Information 1, 2, and 3).

### 2.1. Statistical Analyses

Associations between individual *SOD1* mutations carried by ALS patients and their clinical phenotype were investigated using survival analysis methods. For most patients, we were only able to collect data on two clinically relevant endpoints defined to estimate disease progression: age at symptom onset and age on death (overall survival). Kaplan–Meier curves showing survival for patients stratified by specific *SOD1* mutation or group of mutations (frameshift (FV) and premature stop but nonframeshift variants (nFVs)) were compared using the log-rank test (for two curves) or the chi-square test (for more than two curves). Associations of endpoints with mutation features were analyzed using a Cox proportional hazards regression model. The age of ALS onset was compared between mutations by the Mann–Whitney test. *p* < 0.05 was considered statistically significant. Statistica 13 was used for calculations.

## 3. Results and Discussion

Our dataset comprised 146 individuals (from 54 families) with a total of 38 *SOD1* nonmissense variants predicted to affect the polypeptide sequence. Disease course data were available for 131 patients and 31 mutations. The 38 structural variants presented in Supporting Information 1 were extracted from 47 separate reports (manuscript or personal communication) published between 1997 and 2023. Some reports described single cases and/or families, and others addressed S*OD1* mutations in populations. Despite the fact that the most SOD1 “structural” variants are not deemed pathogenic by ACMG standards, we decided to consider them in our report as pathogenic [19].

Of the 43 reports analyzed, the onset age was not specified in 11 reports, disease duration in 15, symptoms in 22, and familial case status in 9. Clinical course was not reported for 7 variants (p.Thr40_Leu43del, p.Thr89_Asp91del, p.Asp97Metfs^∗^8, p.Leu107_Ser108delinsPro, p.Val120_Gln154delinsGlnLeuLysLysLeuProLys, p.Asp126Thrfs^∗^24, p.Glu133Aspfs^∗^2). Sex was specified for 100 patients: 49 were female and 51 were male.

The median survival time (ALS duration) was 35.5 (range: 6–228) months and the mean survival time was 49 months. No significant difference in survival time was observed between the genders.

The mean age at disease onset for the patients was 46.9 ± 13.3 years (excluding “juvenile” mutations p.Cys112Trpfs^∗^11 and p.Val119del), comparable to all *SOD1* mutant patients from different populations (China: 43.92 ± 9.24 and 46 ± 11.4 years in two reports, North America: 49.7 ± 12.3 years, Poland: 50.2 ± 10.6 years) and about 15 years younger than for all ALS patients (China: 55.5–58.76 years, Europe: 62.1–66.3 years) [20–24].

All the 38 *SOD1* nonmissense variants analyzed here (Supporting Information 1 and 2) were recognized by authors as pathogenic, although their pathogenicity was not confirmed by an in vitro study or cosegregation with ALS.

Most reported mutations affect the distal part of the protein encoded by Exon 4 and Exon 5; among them are three splicing mutations in Intron 4. Only five mutations affect the proximal part of the protein. Four mutations disrupting splicing are located in introns (one in Intron 3 and three in Intron 4). Four mutations are located in the proximal part of the protein encoded by Exons 1 and 2. There are 10 point mutations that introduce a premature stop codon (*n* = 8) or create a splice site (*n* = 2), four insertions or duplications that increase the length of the polypeptide, and the remaining ones result in FVs and a premature stop codon. Two mutations, p.Trp33^∗^ and p.Val30Aspfs^∗^, introduce a stop codon before Greek Key 1 (GK1); p.Lys79_Arg80insSerIle affects the zinc-binding site, seven mutations affect the protein before the electrostatic loop, and 15 mutations affect the loop itself.

Among the 195 variants in *SOD1* identified in ALS patients according to the Amyotrophic Lateral Sclerosis Online Database (ALSoD) (https://alsod.ac.uk/output/gene.php/SOD1#variantsaccession, date: April 17, 2024), point mutations constitute the majority (*n* = 167) and the rest (*n* = 28, 14% of all mutations) are deletions, insertions, and intronic substitutions modifying the polypeptide length by FV, alternative splicing, or premature translation termination [22]. Five of those structural variants (p.Val6CysfsTer4, p.Glu50GlyfsTer39, p.Leu68GlufsTer19, p.Asp97MetfsTer8, and p.Ter155SerextTer6—stop lost) are also reported in the Genome Aggregation Database (gnomAD). The p.Glu50GlyfsTer39 variant is reported as of uncertain significance in a patient with ALS Type 1, and p.Asp97MetfsTer8 was previously reported [25].

Most of the variants analyzed in this study were detected by Sanger sequencing focusing exclusively on the *SOD1* gene. Therefore, any mutations in other genes could affect the interpretation of the pathogenicity of the *SOD1* mutants. Notably, co-occurrence of more pathogenic variants is observed in neurodegenerative disease. For example, *C9orf72* hexanucleotide repeat expansion (HRE) was present in 1.2% of frontotemporal dementia subjects with other pathogenic mutations [26]. Some ALS patients with mutations in *TARDBP*, *PRPH*, *ANG*, *SOD1*, *FUS*, *VAPB*, *DAO*, *UBQLN*, or *OPTN* also carry *C9orf72* HRE. A co-occurrence of *C9orf72* and *SOD1* mutations (p.Asp91Ala and p.Asp111Tyr) has been reported in fALS patients [27]. In the present dataset, only the ALS patient with SOD1 p.Glu134^∗^ was reported to carry another pathogenic variant (*C9orf72* HRE). Each of these mutations could cause ALS on its own, or their effect could overlap and/or enhance each other, as suggested by the early onset at the age of 45 and rapid disease progression. Despite these two identified potentially dominant mutations, the patient has no family history of ALS, which makes it worth studying the relation of two independent potentially causative mutations in more detail, preferably in a mouse model [28]. The presence of two mutations in genes associated with the disease suggests the need for extensive molecular analyses, but also does not undermine the pathogenicity of either of them.

Most of the ALS patients analyzed here were heterozygous for the *SOD1* mutations, with several exceptions. Thus, the p.Gly28_Pro29del mutation, identified in a family of Filipino origin, caused ALS when homozygous but showed low penetrance in heterozygotes. In heterozygous carriers, it was less severe, with the onset delayed by a decade and prolonged duration [17]. Mutations other than the deletion of two amino acids in the conserved portion of SOD1 Loop II enhance naturally occurring alternative splicing of Exon 2 of *SOD1* mRNA. Zinman et al. suggest that alternative splicing, which results in reduced expression of the mutant allele, is responsible for the low penetrance of the mutation [17]. Four other homozygous variants causing null SOD1 activity are associated with the infantile SOD1 deficiency syndrome (iSODDES) identified in children of consanguineous parents [15, 29–31]. Heterozygous carriers do not develop the clinical ALS phenotype.

To date, only nine variants have been identified in the homozygous state (Leu84Phe, Asn86Ser, Asp90Ala, Leu117Val, Leu126Ser, Leu144Ser, p.Gly28_Pro29del, p.Cys112Trpfs^∗^11, and p.Val119del) [32]. Homozygous Asn86Ser, Leu144Ser, p.Gly28_Pro29del, p.Cys112Trpfs^∗^11, and p.Val119del were identified in children with a severe form of early-onset and rapidly progressive ALS [33]. While individuals homozygous for *SOD1* missense mutations cause a severe form of ALS, heterozygous carriers from the same family remain neurologically healthy, develop early-onset ALS (e.g., Asn86Ser and Leu144Ser) or late-onset ALS (e.g., Asp90Ala) suggesting low penetrance of the mutations or the presence of protective factors [33–35]. For the p.Cys112Trpfs^∗^11 or p.Val119del, heterozygous carriers do not develop the ALS phenotype. This contrast suggests that missense mutations are somehow more pathogenic in the heterozygous state than either of these two nonmissense ones but simultaneously less severe in the homozygous state. Biallelic loss of function could result in a novel phenotype with a recessive mode of inheritance, distinct from the typical adult-onset ALS caused by SOD1 toxicity through a gain of function. Indeed, the authors confirmed that the SOD1 protein and activity were essentially absent in the homozygous p.Cys112Trpfs^∗^11 and p.Val119del probands, with activities in heterozygous parents reduced to about half of the controls.

Most of the mutations analyzed were private ones, identified in single carriers or single families (see Supporting Information 1). Only p.Cys112Trpfs^∗^11, p.Val119_Val120insPheLeuGln, and p.Glu134del were identified in more than two families. Of the mutations with patient data available, 26% (*n* = 10) were identified in sporadic cases only, 60% (*n* = 23) in familial cases; p.Glu134del was found in a sporadic and familial case, and three (p.Thr40_Leu43del, p.Asp97Metfs^∗^8, and p.Val119Lysfs^∗^5) were without information. ALS patients with *SOD1* mutations present with a highly heterogeneous phenotype that is clinically indistinguishable between sALS and fALS.

Despite the obvious usefulness of a reliable pathogenicity determination (crucial for a clinical application), most variants analyzed in this study have not been investigated using the ACMG criteria [36, 37]. Numerous reports were in fact published before the ACMG criteria were introduced, but ALS-specific guidelines are also needed to allow a more adequate classification of the large number of variants of uncertain significance [38]. We have not attempted to analyze those variants using the ACMG criteria in light of the above caveats and a lack of conclusive data, but rather considered all reported variants as pathogenic and therefore did not exclude any of them from this study, which could constitute its limitation and should be taken into account in further studies.

One of the features that suggest pathogenicity of variants (often the only one available) is cosegregation with disease in multiple affected families, especially identified in well-established genes such as *SOD1*. Analysis of cosegregation for late-onset neurodegenerative diseases is often impossible to analyze the cosegregation due to the lack of suitable samples from previous generations; therefore, a positive family history of the disorders can be used as indirect evidence of the pathogenicity of the variant in a well-established gene. Identification of the same variant in more than one affected family strengthens the argument. In the 93 fALS cases from 34 families studied here, 24 *SOD1* variants were found. Nine variants were present in more than one independent family, with five of these mutations found in different countries (see Supporting Information 1). All of these cases had a family history. p.Glu134del was identified in six independent patients, with four being sporadic.

Eight mutations were each found in two different populations, but it is impossible to determine whether they arose independently or spread by migration. The p.Glu134^∗^ mutation identified in patients from China, Germany, and the United States is the result of at least two different changes at the nucleotide level (China—c.400G>T and Germany—c.396_399dup, United States—not specified).

### 3.1. Associations of Clinical Endpoints With Individual Mutations

Statistical analysis was performed on 29 *SOD1* mutations (excluding two homozygous mutations causing juvenile ALS: p.Cys112Trpfs^∗^11 and p. Val119del) found in 128 individuals from 44 families. Due to a lack of clinical data, it was not possible to stratify patients by clinical phenotype (e.g., site of onset or personality changes, psychiatric symptoms suggestive of FTD) or clinically relevant endpoints (e.g., wheelchair use or respiratory failure). However, survival analysis of age at onset, age at death, and overall disease duration from onset to death were informative. Only pooled family data were available for five mutations and 28 patients (p.Val30Aspfs^∗^8 (*n* = 12), p.Val119_Val120insPheLeuGln (*n* = 2), p.Val119_Val120insPheLeuGln (*n* = 2), p.Gly28_Pro29del (*n* = 8), and p.Lys129Glyfs^∗^6 (*n* = 4)).

When patients were considered individually, mean data from the relevant family was assigned to each member of the family. Generally, earlier age of onset turned out to be a significant, strong factor for earlier age of death (*HR* = 0.725 per year, 95% CI: 0.725–0.804, *p* = 3 × 10^−24^). A significant difference (*p* < 10^−7^) was found between FVs and nFVs for all analyzed variables: protein length, first affected amino acid, number of affected SOD1 structures, as well as clinical data: age of onset and age of death (Table 1, Supporting Information 2). The year of publication was significantly different between the two groups (median year 2012 for FV and 2018 for nFV, *p* = 0.0051). FV significantly increased the risk that carriers will develop ALS (*HR* = 2.553, 95% CI: 1.722–3.786, *p* = 3.09 × 10^−6^). Because the FV and nFV groups were significantly different as regards most crucial variables, further statistical analyses were performed for each group separately.

In the nFV group, the p.Gly28_Pro29del mutation affecting the v-loop of SOD1 was the least severe (*HR* = 0.224, 95% CI: 0.069–0.727, *p* = 0.013) compared with all other nFVs as regards early ALS onset, and indeed the presence of homozygous patients further confirms this finding. On the other extreme was the p.Lys79_Arg80insSerIle mutant affecting the zinc-binding domain, found to be the strongest predictor of early ALS onset, but this conclusion should be considered with caution since only a single patient with this mutation has been reported. The distance in the protein sequence of the first affected amino acid from the N-terminus of SOD1 was positively associated with an earlier development of ALS at a borderline statistical significance (*HR* = 1.008 per one amino acid, 95% CI: 1.000–1.016, *p* = 0.051) and with shorter mean disease duration (*HR* = 1.008, 95% CI: 1.000–1.016, *p* = 0.04). The HR value of only 1.008 seems to be extremely close to 1, but it should be stressed that it is calculated per one amino acid, counting from the SOD1 N-terminus. Thus, a mutation further downstream in the protein sequence by, for example, 10 amino acids would increase the risk of earlier ALS onset and of shorter survival with ALS by a nontrivial 8%.

**Figure 1:**
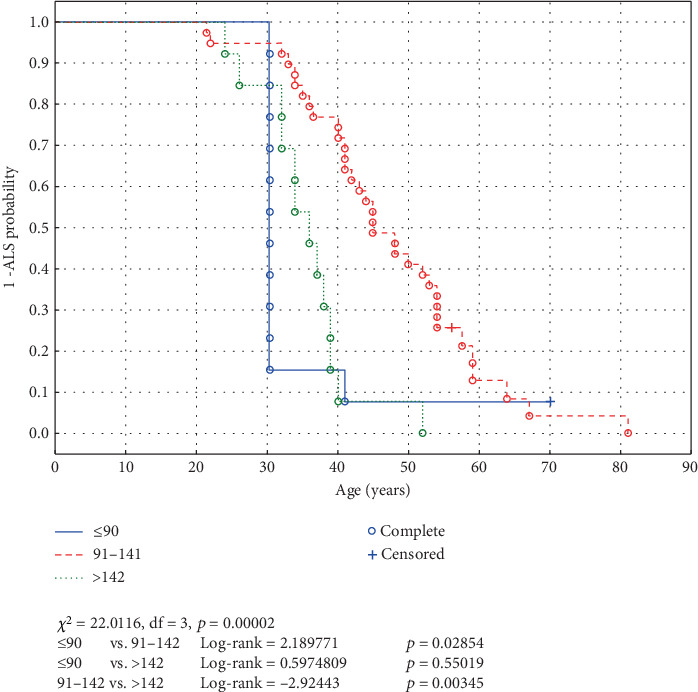
Age of onset of ALS in patients with frameshift mutations affecting different parts of SOD1 protein (< 90 AA, 91–141, or > 142 AA).

In the FV group, mutations affecting the electrostatic loop close to the C-terminus of SOD1 were associated with later ALS onset (*HR* = 0.393, 95% CI: 0.203–0.763, *p* = 0.0057) and were also associated with a longer survival from ALS onset to death (*HR* = 0.266, 95% CI: 0.131–0.540, *p* = 0.00024). Interestingly, variants close to either the N- (AA 1–90, log‐rank = 2.189771, *p* = 0.02854) and C-terminus (AA > 142, log‐rank = 2.92443, *p* = 0.00345) of SOD1 were risk factors of earlier ALS onset compared with the mutations affecting the middle part of the protein (AA 91–142, see Figure 1). This feature is easily explained in the case of the proximal mutations but not for distal ones: A frameshift mutation close to the N-terminus of the protein affects a larger section of the polypeptide sequence than a more distal mutation. One can only speculate that an SOD1 molecule with most of its amino acid sequence, and plausibly also tertiary structure, identical with a native one and a disturbed relatively small C-terminus part could be more “toxic” than the mutant disturbed in most of its C-terminus region.

The risk factor for early death age was FV, and in this group, the mutation in the electrostatic loop (affecting the C-terminus of SOD1) was a protective factor.

No difference in survival time from ALS diagnosis was associated with patients' sex or family history of the disease (fALS vs. sALS).

Frameshift mutations occur in a finite number of protein sequence C-termini combinations. In our data set, 16 FVs occur in 11 different C-termini motifs, with HisThrGlyGlyPro (HTGGP) common for three mutations: p.(Val98Leufs^∗^17), p.Ser108Leufs^∗^15, p.Cys112Trpfs^∗^11, and GlnArgTrpLys (QRWK) for four mutations: p. Leu127Glyfs^∗^6, p.Leu127SerfsTer7^∗^, p.Lys129Glyfs^∗^6, and p.Asn132Glnfs^∗^5. We did not observe any significant difference in clinical data (age of onset and age of death) compared to mutations that do not impact the protein sequence on C-terminus.

This study analyzes for the first time a comprehensive dataset of age at onset, age at death, and disease duration in ALS patients with nonmissense *SOD1* mutations. Sex or familial disease history did not affect ALS onset, disease duration, or age of death. In contrast, different mutations were associated with different ages of symptom onset and death. We provide evidence that through adult life without symptom onset ranging from 21.4 to late 84 years (mean: 48.4), the shift toward younger ages of onset is evident in the penetrance in individuals with nonmissense *SOD1* mutation compared to ALS patients without identified mutation in associated genes, and is comparable to ALS with missense *SOD1* mutations.

Although *SOD1* mutations are generally considered fully penetrant, we observed incomplete penetrance more often than in missense *SOD1* mutations, which might be age related; however, this observation needs further investigation.

Guissart et al. [39] showed that all pathogenic premature termination codons (PTCs) described so far in *SOD1* can theoretically escape nonsense-mediated mRNA decay (NMD), resulting in the production of a truncated protein, supporting the hypothesis that haploinsufficiency is not an underlying mechanism of SOD1 mutant-associated ALS. Those authors found that even when there is massive degradation of mRNA harboring a PTC in the NMD-triggering region, a small residual amount of mutant mRNA could still be detected. Here, we did not stratify the dataset according to the likelihood of NMD. Guissart and colleagues also suggested that PTCs found in NMD-triggering regions are not pathogenic, but such variants have in fact been identified in ALS patients (e.g., p.W33^∗^). For all patients with nonmissense mutations for which SOD1 activity has been determined, its level was decreased, similarly to missense *SOD1* mutations [40]. For at least two homozygous mutations (p.Val119del and p.Cys112Trpfs^∗^11), a complete loss of *SOD1* function was observed [11, 24, 30]. These findings suggest SOD1 haploinsufficiency as a pathological factor, at least coexisting with gain of function.

The relationship between gain and loss of function of SOD1 in the pathomechanism of ALS should be further investigated. First, the ALS-causing mutations that cause gain of function must also cause loss of function of SOD1. On the other hand, there are patients who have ALS due to mutations that cause only loss of SOD1 function. These seemingly contradictory mechanisms can coexist, either attenuating or enhancing each other depending on a time point in the biological process or environmental factors. Our results highlight the need for functional studies of mutations that result in truncated SOD1 protein.

Among the 38 mutations studied here, four were intron ones affecting splicing: c.240-7T>G (p.Lys79_Arg80insSerIle), c.358-304C>G (p.Val120_Gln154delinsGlnLeuLysLysLeuProLys), c.358-11A>G (p.Val120_Gln154delinsPhePheThrGly), and c.358-10T>G (p.Val119_Val120insPheLeuGln). All of them were associated with fALS and considered as pathogenic mainly because of the availability of extensive family trees with access to samples allowing the respective authors to prove cosegregation. Since most ALS patients are sporadic and most familial cases offer only a limited sample access, it seems plausible that numerous ALS cases of undetermined origins could be explained by such mutations, which are routinely omitted in most analyses as variants of uncertain significance. This implies the need to report and investigate all variants, including those in the noncoding regions.

Family history was reported for 35 out of the 38 mutations with clinical information provided. In the 23 mutations (66%), and 123 of 140 cases, 88% were described as causal for fALS, which is comparable to that reported for all SOD1 mutation carriers worldwide (84%) [41]. This supports the true pathogenicity of these mutations, but this shift toward familial cases may indicate that in sporadic cases *SOD1* mutations could be underreported. It is likely that in the case of identified *SOD1* variants, especially the sporadic ones, the same mechanism can be observed as in the case of published negative results. This is even more likely if one considers that the absence of a family history may be due to incomplete penetrance, inadequate recording, or small family size, which may mask familiarity. At the same time, the low penetrance of the mutation in our dataset is noted, for example, p.Gly28_Pro29del and p.Glu134del mutations were identified in both sporadic and familial cases. To understand the pathomechanism of *SOD1* mutations, it is crucial to encourage both scientists and publishers to publish case reports that do not necessarily prove pathogenicity.

Individual *SOD1* nonmissense mutations show differences between them in terms of age at symptom onset, age at death, or disease duration, which are also observed in missense *SOD1* variants. The underlying mechanism remains unclear but consistent disease course in different families or environments (same mutations identified in different countries and continents) may lean towards the nature of the individual mutation itself.

*SOD1* mutations have been annotated variably in the literature, complicating comparison of carriers of the same mutation across reports. Most of the mutations were published without counting the first methionine, but we also observed errors in the predicted protein consequence (e.g., the HGVS Nomenclature p.Leu107_Ser108del vs. the literature nomenclature S105deltaSL, the HGVS Nomenclature p. Gly130Trpfs^∗^3 vs. the literature nomenclature p.Lys129fsTer5 or Lys128 fsX131). Our study organizes protein nomenclature according to the HGVS Nomenclature recommendation inferred from reported cDNA annotation or an attached electrophoretogram of Sanger sequencing.

Nonmissense variants account for 14% of the 165 *SOD1* variants affecting the protein sequence reported in the gnomAD (April 17, 2024) (frameshift *n* = 11, inframe deletion *n* = 4, stop gained *n* = 5, start lost *n* = 1, stop lost *n* = 2, missens = 144), which is slightly less than among the variants identified in ALS patients (Supporting Information 4) [42]. Those nonmissense variants are rare (identified in 1 up to 11 individual subjects, the latter in the case of p.Ter155SerextTer6) and annotated as heterozygous. p.Trp33^∗^, p.Asp97Metfs^∗^8, p.Leu107_Ser108del, p.Asp126Thrfs^∗^24, and p.Glu134del have also been reported in ALS patients. The presence or absence of a variant in the gnomAD does not indicate its pathogenicity, nor does it mean that variants will not be identified in ALS patients, as the gnomAD contains whole exome and whole-genome sequences aggregated from many large-scale sequencing projects. Well-established ALS pathogenic variants such as p.Ala5Val are also present in the gnomAD. Noteworthy, there are rare variants that most likely affect splicing in the gnomAD that have also been identified in ALS patients but (with a few exceptions) were not further studied in the context of ALS.

## 4. Limitation of the Study

The present study has obvious limitations common to similar projects, such as retrospective data collection which weakens its general conclusions [43]. These limitations include nonstandardized age of onset and disease course, a relatively small group of patients with individual mutations, incomplete information about their phenotype and family history, and probably the most important one, unaccounted for coexisting potentially pathogenic variants in other genes associated with ALS.

## 5. Conclusions

Nonmissense *SOD1* variants constitute a substantial proportion of all the *SOD1* variants identified in ALS patients. Our study highlights the necessity of including nonmissense *SOD1* mutation carriers in clinical trials and research studies to help elucidate the role of individual domains of the SOD1 protein in ALS pathogenicity. Many of the variants introducing a premature stop codon have been proposed to cause ALS via a loss-of-function mechanism, but the present study indicates that both loss- and gain-of-function mechanisms are likely involved in ALS pathology.

## Figures and Tables

**Table 1:** Survival analysis of associations between ALS endpoints and nonmissense SOD1 mutation features with univariate Cox proportional hazards regression model.

**Endpoints**	**All ALS patients**					**Patients with nonframeshift variants (nFVs)**					**Patients with frameshift variant (FV)**				
	**Risk factors**	**HR**	**−95% CI**	**+95% CI**	**p**	**Risk factors**	**HR**	**−95% CI**	**+95% CI**	**p**	**Risk factors**	**HR**	**−95% CI**	**+95% CI**	**p**
Age of onset or last known without ALS	Frameshift	1.905	1.258	2.886	0.002337938	v_loop	0.228	0.070	0.741	0.013932	e_loop	0.227	0.111	0.465	5.17*e* − 05
	v_loop	0.236	0.073	0.766	0.01622463	Zincbind	9.262	1.081	79.345	0.042239	c_gkh	22.432	6.663	75.522	5.14*e* − 07
	Zincbind	2.253	1.201	4.228	0.011441005										

Age of death or last known as alive	Frameshift	2.310	1.496	3.568	0.00016001	Zincbind	27.833	2.524	306.923	0.006611	Zincbind	1.074	0.475	2.426	0.863646
	Zincbind	2.055	0.973	4.339	0.058923244	First aa	1.014	1.005	1.023	0.002489	e_loop	0.348	0.167	0.723	0.004699
	First aa	1.013	1.005	1.020	0.000943985										
	e_loop	0.376	0.222	0.638	0.000281164										
	First aa	1.020	1.013	1.027	1.41264*e* − 08										

Mean disease duration (months)	First aa	1.014	1.008	1.020	8.37338*e* − 06	First aa	1.008	1.000	1.016	0.040899	First aa	1.020	1.010	1.029	5.17*e* − 05

Abbreviations: 95% CI: 95% confidence interval; c_gkh: mutations with CGH motif on protein's C-terminus; e_loop: mutations affect the electrostatic loop; first aa: first affected amino acid; HR: hazards ratio; v_loop: mutations affected v-loop; zincbind: mutations affect the zinc-binding site.

## Data Availability

Data sharing is not applicable to this article, as no new data were created or analyzed in this study.

## Supporting Information

Supporting Information 1 Table S1: List of SOD1 nonmissense mutations identified in ALS patients.

Supporting Information 2 Table S2: Clinical patients' data.

Supporting Information 3 Table S3: Mutated protein sequences.

Supporting Information 4 Table S4: Nonmissense SOD1 reported in the Genome Aggregation Database (gnomAD) (https://gnomad.broadinstitute.org/gene/ENSG00000142168?dataset=gnomad_r4).
