# Supplementary material for: Phenotypic Characterization of ALS-Causing SOD1 Mutations Affecting Polypeptide Length
Source: Hum Mutat. 2025 Jun 16;2025:9792233. doi: 10.1155/humu/9792233 (PMC12185200; doi:10.1155/humu/9792233)
Supplement: Supporting Information 1 — Table S1: List of SOD1 nonmissense mutations identified in ALS patients. [file 9792233.f1.docx]

Supplementary Table 1. List of *SOD1* non-missense mutations identified in ALS patients (published in peer-reviewed journals, oral or poster presentations at conferences, or reported personally to P.M.A.)

|  | coding DNA reference sequence ENSP00000270142.7: | Protein reference sequence ENST00000270142.11: | Protein variant (original article nomenclature) | Familial/Sporadic |  | Patient origin (Living place) | families/patients |  |
| --- | --- | --- | --- | --- | --- | --- | --- | --- |
| 1 | c.37_54dup | p.Gly13_Ile18dup | p.19_24insGPVQGI | fALS | heterozygous | Japan | 1/5 | 1 |
| 2 | c.83_88del | p.Gly28_Pro29del | G27delGP | fALS | heterozygous, homozygous | Phillipino (Canada), USA | 2/3 | 2,3 |
| 3 | c.88_89insA | p.Val30Aspfs*8 | V29DEGVGKHX | fALS | heterozygous | China | 1/1 | 4 |
| 4 | c.98G>A | p.Trp33* | W32X, W32* | sALS | heterozygous | Poland, Germany | 2/2 | 5, 6 |
| 5 | c.118_129del | p.Thr40_Leu43del | L38delLTEG |  | heterozygous | Germany | 1/1 | a |
| 6 | c.240-7T>G | p.Lys79_Arg80insSerIle | E78_R79insSI | fALS | heterozygous | Switzerland | 1/1 | 7 |
| 7 | c.266_274del | p.Thr89_Asp91del | T88delTAD |  | heterozygous | USA | 1/1 | 8,9 |
| 8 | c.273_275dup | p.Asp91_Lys92insAsn | D91_K92insN | fALS | heterozygous | China | 1/9 | 10 |
| 9 | c.275_276del | p.Lys92Argfs*9 | Lys91ArgfsTer8 | sALS | heterozygous | Hungry | 1/1 | 9,11 |
| 10 | c.289del | p.Asp97Metfs*8 | D96Mfs*8 |  | heterozygous | Sweden (Denmark) | 1/1 | 12 |
| 11 | c.292_314del | p.(Val98Leufs*17) | p.Val98LeufsTer17 | sALS | heterozygous | Italy | 1/1 | 13 |
| 12 | c.319_324del | p.Leu107_Ser108del | S105deltaSL | sALS | heterozygous | Hispanic (USA) | 1/1 | 8 |
| 13 |  | p.Leu107_Ser108delinsPro |  | fALS | heterozygous | Spain | 1/1 | 14 |
| 14 | c.320dup | p.Ser108Leufs*15 | Ser108LeufsTer15; p.Leu106 fs*15 | sALS | heterozygous | Italy | 1/1 | 9,15 |
| 15 | c.335dup | p.Cys112Trpfs*11 |  | sALS | homozygous | Lebanon (Brasil), Afganistan | 2/2 | 16–18 |
| 16 | c.355delinsAAAAC | p.Val119Lysfs*5 | V118KTGPX / p.Val119Lysfs*5 |  | heterozygous | United Kingdom | 1/1 | 19 |
| 17 | c.357_357+2del | p.Val119del | c.357_357+2delGGT | sALS | homozygous | Arab Muslim origin | 1/1 | 20 |
| 18 | c.358-304C>G | p.Val120_Gln154delinsGlnLeuLysLysLeuProLys | V118insQLKKLPKX | fALS | homozygous | France | 1/1 | 6,21 |
| 19 | c.358-11A>G | p.Val120_Gln154delinsPhePheThrGly | V118insFFTGX | fALS | heterozygous | U.S.A. | 1/1 | 22 |
| 20 | c.358-10T>G | p.Val119_Val120insPheLeuGln | V118insFLQ | fALS | heterozygous | France, USA | 6/19 | 23–25 |
| 21 | c.376del | p.Asp126Thrfs*24 | D125TWAKVEMKKVQRQETLEVVWLVVX | fALS | heterozygous | USA | 1/1 | 2,6,9 |
| 22 | c.379_380del | p.Leu127Glyfs*6 | L126GQRWKX | fALS | heterozygous | Japan | 3/12 | 26–28 |
| 23 | c.380T>A | p.Leu127* | L126*, L126Z | fALS | heterozygous | Poland, USA | 2/6 | 5,22 |
| 24 |  | p.Leu127SerfsTer7* |  | fALS | heterozygous | Spain | 1/1 | 14 |
| 25 | c.380_383dup | p.Lys129Glyfs*6 | G127GGQRWKX, G127X, Lys127insTGGG | fALS | heterozygous | Denmark | 1/3 | 29,30 |
| 26 | c.383_392dup | p.Asn132Glnfs*5 | p.N132Qfs*5 | fALS | heterozygous | China | 2/4 | 31,31 |
| 27 | c.387_388del | p.Gly130Trpfs*3 | p.Lys129fsTer5 | fALS | heterozygous | China | 1/1 | 32,33 |
| 28 | c.389_400del | p.Gly130_Glu133del | G129delGGNE | fALS | heterozygous | Italy | 1/1 | 34 |
| 29 | c.390_391del/c.389_390del | p.Gly131Lysfs*2 | p.Gly131fs | fALS | heterozygous | China | 1/1 | 35 |
| 30 | c.397G>T | p.Glu133* | p.Glu133Ter | sALS | heterozygous | China | 1/1 | 32 |
| 31 | c.398_399insTT | p.Glu133Aspfs*2 | E132DX | fALS | heterozygous | United Kingdom | 1/1 | 36 |
| 32 | c.396_399dup | p.Glu134*+C9orf72 | E133X +c9orf72 | sALS | heterozygous | Germany | 1/1 | 37  b |
| 33 | c.400_402del | p.Glu134del | E133deltaE | fALS, sALS | heterozygous | Italy, Spain, USA | 6/7 | 13,25,38–40 |
| 34 | c.400G > T | p.Glu134* | p.E134* | fALS | heterozygous | China | 1/1 | 41 |
| 35 | c.409A>T | p.Lys137* | K136X | sALS | heterozygous | Italy | 2/6 | 9,15 |
| 36 | c.424G>T | p.Gly142* | G141X | fALS | heterozygous | USA | 2/6 | 8,9,42 |
| 37 | c.435delinsCGTTTA | p.Leu145Phefs*3 | L144FVX | fALS | heterozygous | Japan | 1/1 | 9,43 |
| 38 | c.441T>A | p.Cys147* | C146X | fALS | heterozygous | Bulgaria, China | 2/13 | 9,44  c |

**Reference**

a. Unpublished PMA

b. Unpublished PMA

c. Unpublished PMA

1. Nakamura, A. et al. Slowly progressing lower motor neuron disease caused by a novel duplication mutation in exon 1 of the SOD1 gene. Neurobiol. Aging 35, 2420.e7-2420.e12 (2014).

2. Brown, J. A. et al. SOD1, ANG, TARDBP and FUS mutations in amyotrophic lateral sclerosis: A United States clinical testing lab experience. Amyotroph. Lateral Scler. 13, 217–222 (2012).

3. Zinman, L. et al. A mechanism for low penetrance in an ALS family with a novel SOD1 deletion. Neurology 72, 1153–1159 (2009).

4. Hu, J. et al. A novel SOD1 mutation in amyotrophic lateral sclerosis with a distinct clinical phenotype. Amyotroph. Lateral Scler. Off. Publ. World Fed. Neurol. Res. Group Mot. Neuron Dis. 13, 149–154 (2012).

5. Berdyński, M. et al. SOD1 mutations associated with amyotrophic lateral sclerosis analysis of variant severity. Sci. Rep. 12, 103 (2022).

6. Keskin, I. et al. Comprehensive analysis to explain reduced or increased SOD1 enzymatic activity in ALS patients and their relatives. Amyotroph. Lateral Scler. Front. Degener. 18, 457–463 (2017).

7. Birve, A. et al. A novel SOD1 splice site mutation associated with familial ALS revealed by SOD activity analysis. Hum. Mol. Genet. 19, 4201–4206 (2010).

8. Andersen, P. M. et al. Sixteen novel mutations in the Cu/Zn superoxide dismutase gene in amyotrophic lateral sclerosis: a decade of discoveries, defects and disputes. Amyotroph. Lateral Scler. Mot. Neuron Disord. Off. Publ. World Fed. Neurol. Res. Group Mot. Neuron Dis. 4, 62–73 (2003).

9. Guissart, C. et al. Premature termination codons in SOD1 causing Amyotrophic Lateral Sclerosis are predicted to escape the nonsense-mediated mRNA decay. Sci. Rep. 10, 20738 (2020).

10. Li, Y. et al. A novel D90_K91insN mutation in exon 4 of the SOD1 gene caused familial amyotrophic lateral sclerosis in a Chinese pedigree. Amyotroph. Lateral Scler. Front. Degener. 19, 516–521 (2018).

11. Tripolszki, K. et al. Genetic analysis of the SOD1 and C9ORF72 genes in Hungarian patients with amyotrophic lateral sclerosis. Neurobiol. Aging 53, 195.e1-195.e5 (2017).

12. Forsgren, E. Using Patient-Derived Cell Models to Investigate the Role of Misfolded SOD1 in ALS. (Pharmacology and Clinical Neuroscience, Umeå University, Umeå, 2017)

13. Theme 02 - Genetics and Genomics. Amyotroph. Lateral Scler. Front. Degener. 23, 40–56 (2022).

14. Vázquez-Costa, J. F. et al. Characterizing SOD1 mutations in Spain. The impact of genotype, age, and sex in the natural history of the disease. Eur. J. Neurol. (2022) doi:10.1111/ene.15661.

15. Canosa, A. et al. A novel p.Ser108LeufsTer15 SOD1 mutation leading to the formation of a premature stop codon in an apparently sporadic ALS patient: insights into the underlying pathomechanisms. Neurobiol. Aging 72, 189.e11-189.e17 (2018).

16. de Souza, P. V. S. et al. Progressive spastic tetraplegia and axial hypotonia (STAHP) due to SOD1 deficiency: is it really a new entity? Orphanet J. Rare Dis. 16, 360 (2021).

17. Andersen, P. M. et al. Phenotype in an Infant with SOD1 Homozygous Truncating Mutation. N. Engl. J. Med. 381, 486–488 (2019).

18. Park, J. H. et al. SOD1 deficiency: a novel syndrome distinct from amyotrophic lateral sclerosis. Brain J. Neurol. 142, 2230–2237 (2019).

19. Jackson, M. et al. Copper/zinc superoxide dismutase 1 and sporadic amyotrophic lateral sclerosis: analysis of 155 cases and identification of a novel insertion mutation. Ann. Neurol. 42, 803–807 (1997).

20. Ezer, S. et al. Infantile SOD1 deficiency syndrome caused by a homozygous SOD1 variant with absence of enzyme activity. Brain awab416 (2021) doi:10.1093/brain/awab416.

21. Valdmanis, P. N. et al. A Mutation that Creates a Pseudoexon in SOD1 Causes Familial ALS. Ann. Hum. Genet. 73, 652–657 (2009).

22. Zu, J. S. et al. Exon 5 encoded domain is not required for the toxic function of mutant SOD1 but essential for the dismutase activity: identification and characterization of two new SOD1 mutations associated with familial amyotrophic lateral sclerosis. Neurogenetics 1, 65–71 (1997).

23. Sapp, P. C. et al. Identification of three novel mutations in the gene for Cu/Zn superoxide dismutase in patients with familial amyotrophic lateral sclerosis. Neuromuscul. Disord. NMD 5, 353–357 (1995).

24. Muratet, F. et al. Impact of a frequent nearsplice SOD1 variant in amyotrophic lateral sclerosis: optimising SOD1 genetic screening for gene therapy opportunities. J. Neurol. Neurosurg. Psychiatry 92, 942–949 (2021).

25. Cudkowicz, M. E. et al. Epidemiology of mutations in superoxide dismutase in amyotrophic lateal sclerosis. Ann. Neurol. 41, 210–221 (1997).

26. Watanabe, Y., Kato, S., Adachi, Y. & Nakashima, K. Frameshift, nonsense and non amino acid altering mutations in SOD1 in familial ALS: report of a Japanese pedigree and literature review. Amyotroph. Lateral Scler. Other Motor Neuron Disord. 1, 251–258 (2000).

27. Kadekawa, J. et al. A clinicopathological study of a patient with familial amyotrophic lateral sclerosis associated with a two base pair deletion in the copper/zinc superoxide dismutase (SOD1) gene. Acta Neuropathol. (Berl.) 94, 617–622 (1997).

28. Kato, S. et al. Familial amyotrophic lateral sclerosis with a two base pair deletion in superoxide dismutase 1: gene multisystem degeneration with intracytoplasmic hyaline inclusions in astrocytes. J. Neuropathol. Exp. Neurol. 55, 1089–1101 (1996).

29. Andersen, P. Phenotypic heterogeneity in motor neuron disease patients with CuZn- superoxide dismutase mutations in Scandinavia. Brain 120, 1723–1737 (1997).

30. Jonsson, P. A. et al. Minute quantities of misfolded mutant superoxide dismutase‐1 cause amyotrophic lateral sclerosis. Brain 127, 73–88 (2004).

31. Chen, S. et al. A novel 10-base pair insertion mutation in exon 5 of the SOD1 gene in a Chinese family with amyotrophic lateral sclerosis. Neurobiol. Aging 45, 212.e1-212.e4 (2016).

32. Tang, L., Ma, Y., Liu, X., Chen, L. & Fan, D. Better survival in female SOD1-mutant patients with ALS: a study of SOD1-related natural history. Transl. Neurodegener. 8, 2 (2019).

33. Association between superoxide dismutase 1 mutations and clinical phenotypes in Chinese patients with familial amyotrophic lateral sclerosis | Semantic Scholar. https://www.semanticscholar.org/paper/Association-between-superoxide-dismutase-1-and-in-Zhang-Tang/d532b4601520d7320f1b615af91e96892a0ba8ed.

34. Coppedè, F. et al. Increase in DNA methylation in patients with amyotrophic lateral sclerosis carriers of not fully penetrant SOD1 mutations. Amyotroph. Lateral Scler. Front. Degener. 19, 93–101 (2018).

35. Chen, Y.-P. et al. Role of genetics in amyotrophic lateral sclerosis: a large cohort study in Chinese mainland population. J. Med. Genet. 59, 840–849 (2022).

36. Orrell, R. W. et al. Clinical and functional investigation of 10 missense mutations and a novel frameshift insertion mutation of the gene for copper-zinc superoxide dismutase in UK families with amyotrophic lateral sclerosis. Neurology 48, 746–751 (1997).

37. Witzel, S. et al. Fast versus slow disease progression in amyotrophic lateral sclerosis-clinical and genetic factors at the edges of the survival spectrum. Neurobiol. Aging 119, 117–126 (2022).

38. Gianferrari, G. et al. Case report: p.Glu134del SOD1 mutation in two apparently unrelated ALS patients with mirrored phenotype. Front. Neurol. 13, 1052341 (2022).

39. Hosler, B. A. et al. Three novel mutations and two variants in the gene for Cu/Zn superoxide dismutase in familial amyotrophic lateral sclerosis. Neuromuscul. Disord. 6, 361–366 (1996).

40. Chiò, A. et al. Prevalence of SOD1 mutations in the Italian ALS population. Neurology 70, 533–537 (2008).

41. Chen, L.-X. et al. SOD1 Mutation Spectrum and Natural History of ALS Patients in a 15-Year Cohort in Southeastern China. Front. Genet. 12, (2021).

42. Nakamura, M. et al. A truncating SOD1 mutation, p.Gly141X, is associated with clinical and pathologic heterogeneity, including frontotemporal lobar degeneration. Acta Neuropathol. (Berl.) 130, 145–157 (2015).

43. Kawamata, C., Morita, M., Shibata, N. & Nakano, I. [Familial amyotrophic lateral sclerosis (FALS) with a novel SOD1 gene mutation: a clinicopathological study]. Rinsho Shinkeigaku 47, 211–216 (2007).

44. Wu, J., Shen, E., Shi, D., Sun, Z. & Cai, T. Identification of a novel Cys146X mutation of SOD1 in familial amyotrophic lateral sclerosis by whole-exome sequencing. Genet. Med. Off. J. Am. Coll. Med. Genet. 14, 823–826 (2012).
