# Supplementary material for: Phenotypic Characterization of ALS-Causing SOD1 Mutations Affecting Polypeptide Length
Source: Hum Mutat. 2025 Jun 16;2025:9792233. doi: 10.1155/humu/9792233 (PMC12185200; doi:10.1155/humu/9792233)
Supplement: Supporting Information 4 — Table S4: Nonmissense SOD1 reported in the Genome Aggregation Database (gnomAD) (https://gnomad.broadinstitute.org/gene/ENSG00000142168?dataset=gnomad_r4). [file 9792233.f4.docx]

Supplementary Table 5. Non-missense SOD1 reported in The Genome Aggregation Database (gnomAD) (<https://gnomad.broadinstitute.org/gene/ENSG00000142168?dataset=gnomad_r4>).

| HGVS Consequence | Transcript Consequence | VEP Annotation | Allele Frequency |
| --- | --- | --- | --- |
| p.Met1? | c.2T>C | start_lost | 0.0000012003360941063497 |
| p.Val6CysfsTer4 | c.15del | frameshift_variant | 0.0000015913025766371322 |
| p.Ile19del | c.56_58del | inframe_deletion | 0.0000012003303309070656 |
| p.Asn20IlefsTer11 | c.59del | frameshift_variant | 6.843315449468958e-7 |
| p.Glu25Ter | c.73_77del | splice_acceptor_variant | 0.000001244459046097252 |
| p.Gly28AspfsTer3 | c.83del | frameshift_variant | 6.853060782537305e-7 |
| p.Trp33Ter | c.98G>A | stop_gained | 0.0000020535876861406106 |
| p.Glu41ArgfsTer10 | c.120_121insA | frameshift_variant | 0.0000012003994929512542 |
| p.Glu50GlyfsTer39 | c.149del | frameshift_variant | 0.00000547564910397847 |
| p.Glu50Ter | c.148G>T | stop_gained | 6.844561379973088e-7 |
| p.Thr55LysfsTer34 | c.164del | frameshift_variant | 0.0000012023537276572619 |
| p.His64GlnfsTer3 | c.191dup | frameshift_variant | 6.847626612616067e-7 |
| p.Leu68GlufsTer19 | c.201_207del | frameshift_variant | 0.000004773102612159956 |
| p.Asp97MetfsTer8 | c.289del | frameshift_variant | 0.0000013681443994324936 |
| p.Leu107_Ser108del | c.319_324del | inframe_deletion | 0.0000015904673747427419 |
| p.Ser106Ter | c.317C>G | stop_gained | 0.0000031809651048128003 |
| p.Ser108Ter | c.323C>G | stop_gained | 0.0000012007396556278667 |
| p.Ala124del | c.371_373del | inframe_deletion | 0.0000015910139531923694 |
| p.Asp126ThrfsTer24 | c.376del | frameshift_variant | 0.00000657133845021554 |
| p.Glu134del | c.400_402del | inframe_deletion | 0.0000012393247662943323 |
| p.Ala141GlyfsTer23 | c.421dup | frameshift_variant | 6.841480167917289e-7 |
| p.Ter155SerextTer6 | c.464A>C | stop_lost | 0.0000068414767638571 |
| p.Ter155TyrextTer6 | c.465A>T | stop_lost | 0.0000024129236189028437 |
